# High plasma uric acid concentration: causes and consequences

**DOI:** 10.1186/1758-5996-4-12

**Published:** 2012-04-04

**Authors:** Erick Prado de Oliveira, Roberto Carlos Burini

**Affiliations:** 1 Center for exercise metabolism and nutrition (CeMENutri), Department of Public Health, Botucatu School of Medicine (UNESP), Botucatu, Brazil; 2 Department of Pathology, Botucatu School of Medicine (UNESP), Botucatu, Brazil; 3 CeMENutri-Faculdade de Medicina, Departamento de Saúde Pública (FMBUNESP), Distrito de Rubião Jr, s/n, 18.618-970 Botucatu, SP, Brazil

**Keywords:** Uric acid, Diet, Protection, Risk, Metabolic syndrome

## Abstract

High plasma uric acid (UA) is a precipitating factor for gout and renal calculi as well as a strong risk factor for Metabolic Syndrome and cardiovascular disease. The main causes for higher plasma UA are either lower excretion, higher synthesis or both. Higher waist circumference and the BMI are associated with higher insulin resistance and leptin production, and both reduce uric acid excretion. The synthesis of fatty acids (tryglicerides) in the liver is associated with the de novo synthesis of purine, accelerating UA production. The role played by diet on hyperuricemia has not yet been fully clarified, but high intake of fructose-rich industrialized food and high alcohol intake (particularly beer) seem to influence uricemia. It is not known whether UA would be a causal factor or an antioxidant protective response. Most authors do not consider the UA as a risk factor, but presenting antioxidant function. UA contributes to > 50% of the antioxidant capacity of the blood. There is still no consensus if UA is a protective or a risk factor, however, it seems that acute elevation is a protective factor, whereas chronic elevation a risk for disease.

## Introduction

High plasma uric acid (UA) is a prerequisite for gout and is also associated with the Metabolic Syndrome and risk factors for cardiovascular diseases [1-4]. Hyperuricemia increase in 16% all causes of mortality and 39% of total cardiovascular disease [5].

The hyperuricemia is defined as ≥7 mg/dL for men and ≥6.0 mg/dL for women [6], and is found mainly in postmenopausal women, African American, patients with renal disease and alcohol intake [7]. Furthermore, many factors can influence the concentrations of UA, eg. diet, obesity, and Metabolic Syndrome [1,8-10]. However, we still don't know if UA is a protective factor for the moderate oxidative stress in these situations or if it's a risk factor.

The aim of this review was to discuss the function of the UA in our organism and the main causes and consequences of the higher concentration of UA.

## Chemical characterization and biological importance

Uric acid (2,6,8 trioxypurine-C5H4N4O3) is an organic compound that is endogenously produced by animals as a purine metabolite. It is formed by the liver and mainly excreted by the kidneys (65-75%) and intestines (25-35%). UA is the end product of purine metabolism in humans due to the loss of uricase activity, which led to humans having higher UA levels than other mammals [11,12].

Due to its double bonds, uric acid has excellent antioxidant capacity, and it can be responsible for 2/3 of total plasma antioxidant capacity [13,14].

Because it is a weak acid that have a high dissociation constant, uric acid circulates in plasma (pH 7.4) predominantly (98%) in the form of a monovalent sodium salt (urate) [15]. It shows low solubility in water (as well as in plasma), and it would theoretically reach plasma saturation in the concentration of 6.4 mg/dL, which may not occur because solubility increase is provided by its binding to proteins, namely albumin, which is its main transporter. Protein-bound uric acid shows plasma solubility that is 70% higher than in its free state [16]. Uric acid pathogenesis is usually associated with gouty arthritis or nephrolithiasis [12].

High uricemia pathogenicity is associated with its low solubility in the extracellular environment leading to crystal formation, low affinity (and deposition) to certain tissues and antigenicity (after crystal phagocytosis). This mixture of quantitative and qualitative etiological hyperuricemia factors is confounding because normouricemic individuals may show symptoms while others with hyperuricemia may not. In the clinical context, hyperuricemia is seen as a prognostic indicator of renal disease, diabetes mellitus, cardiovascular disease and inflammation [7,17-23], thus being a (modest) risk factor for mortality [1].

## Uricemia homeostasis

Physiologically, uric acid plasma concentrations increases with age; they are smaller in women of childbearing age and, in post menopause women, it increase to similar values to those found in males [2,3].

The balance between uric acid production and excretion in males shows a turnover of 600- 700 mg/day for a pool with a maximum of 1,000 mg, as shown by isotopic studies. Uric acid entry into the pool is controlled by hepatic production, which receives endogenous (nucleoproteins) and exogenous (dietary) precursor proteins. Uric acid exit from the pool in males is mainly controlled by the kidneys and by urine-forming factors, renal plasma flow, glomerular filtration and tubular exchange [24,25].

## Excretion control

In the kidney, uric acid and urate are initially filtered and additionally secreted. However, the largest part (90%) is usually reabsorbed and returns to blood [12].

The factors that can influence the uptake of urate by the kidney are: plasma concentrations, volemia and renal plasma flow modulators. At this point, renal excretion of urate follows the same extra-renal limiting factors as those of glomerular filtration. Kidney participation is associated with glomerular filtration (90% filtrate), proximal reabsorption (by an active process) and post-secretory resorption in the distal tubule, ascending loop and collecting duct. In this phase, the renal excretion of urate follows the limiting factors that accompany renal diseases (glomerular and medullary). The urate renal excretion phase is that of post-secretory resorption [24].

Proximal tubular reabsorption of uric acid is competitive with monocarboxylic organic acids, and it can be inhibited by oxalic acid, lactic acid and ketone bodies (acetoacetic and beta-hydroxybutyric acids). Metabolic situations with higher production and circulation of such acids are accompanied by the hypoexcretion of uric acid in urine [24].

Lactic acid is a product of anaerobic glycolysis (NADH-dependent) in alcoholics, resulting from the metabolism of ethanol (due to higher NADH production), and in inflammation/infection (due to PDH inhibition by pro-inflammatory cytokines) [24].

Ketone bodies are produced in the liver by partial oxidation of acetyl coenzyme A due to excessive peripheral lipolysis. Ketonuria occurs in decompensated diabetic individuals and in acute fasting (weight loss by carbohydrate-restricted diets). Factors that increase uric acid urinary excretion: extracellular volume expansion and tubular resorption inhibition [24].

Acute renal failure can also rise UA by decreased of renal excretion [26]. Furthermore, the consumption of some drugs are associated with the raise of serum UA (ciclosporin, ethambutol, pyrazinamide, cytotoxic chemotherapy). On the other hand, other drugs (benzbromarone, losartan, probenecid, sulfinpyrazon) diminishes the UA concentration mainly by the inhibition of a specific anion transporters (URAT1), that is responsible for the reabsorption of renal uric acid, which explain the uricosuric effect of these drugs [2].

## Production control

Purine bases and their nucleotides are rapidly captured by the liver [27] and converted into xanthines, metabolized by uric acid or recycled by salvage pathways or *de novo *synthesis, and the latter requires a higher energetic cost (ATP consumption) [28]. Uric acid formation by xanthine occurs via hypoxanthine by the action of xanthine oxidase [29]. In most mammals, uric acid is converted into allantoin by uricase and later into urea, which are excreted. For this reason, uricemia in these animals corresponds to 10% of human values [30,31].

Diseases accompanied by great cell depletion, such as leukemia, leukocytosis and dystrophies can increase the provision of nucleic acids to the liver and result in a larger production of uric acid. Hence, diseases resulting from innate purine metabolic errors may also result in hyperuricemia [2,3,12].

## Diet

The relation between diet and UA has not yet been fully clarified since most studies have not estimated basal urate concentrations, have not excluded confounding factors or have not even correctly evaluated ingested nutrients [32].

The human diet is very poor in urate, which is produced mainly in the liver and to a less extent in the small intestine. The production of UA depends of purine ingestion [2], however, a purine-rich diet would be responsible for an increasing only in 1 to 2 mg/dL of UA [33,34].

It is recommended that individuals with hyperuricemia should not ingest a large amount of purine-rich food (for instance, veal, bacon, kid meat, mutton, turkey, pork, duck, goose, etc.) [35], and it is suggested that a high intake of meat and seafood are related to increased UA, but the same relation is not valid for total protein intake [36].

During fructose metabolism, the phosphorylation of such monosaccharide into fructose 1-phosphate occurs by means of enzyme fructokinase. Next, enzyme aldolase B breaks fructose 1-phosphate into dihydroxyacetone phosphate (DHAP) and D-glyceraldehyde. When high fructose intake occurs, fructose phosphorylation into fructose 1-phosphate is fast, but the reaction with aldolase is slow. Hence, fructose 1-phosphate accumulates, and inorganic concentrations of intracellular phosphate also decrease. The low availability of phosphate limits ATP formation (ADP + Pi), and ADP or AMP resulting from such metabolism is catabolized, thus leading to hyperuricemia [37].

There are other ways to increase urate concentrations, such as the intake of sorbitol, sucrose, lactate and methylxanthines [38]. After absorption, sorbitol is converted into fructose by the liver, and thus, it can increase UA production [39,40].

Fruit and vegetables, in addition to flavonoids, can contain other nutrients that can affect the plasma total antioxidant capacity (TAC). Lotito & Frei (2006) [40] reported that the TAC increase observed after the intake of these flavonoid-rich food types is not specifically caused by flavonoids because such nutrients are probably highly metabolized after ingestion. Possibly, what would increase TAC would be the UA elevation resulting from fructose metabolism. The mechanism for such elevation would be the degradation of purine nucleotides or UA excretion reduction [41-43].

High vitamin-C intake shows an inverse relation with UA [44] as such vitamin has a uricosuric effect due to competition of UA renal resorption through a change in the anion transportation system in the proximal tubule [45,46].

Beer intake is an independent factor from UA increase [32] both due to its alcoholic content and high-quality purine [47]. Beer intake has a stronger power to increase UA than liqueur, but moderate wine intake does not increase UA [36,48]. Alcohol intake increases UA concentrations by reducing excretion [49,50] and increasing urate production [51].

Dairy product intake has an inverse relation with UA [32,36,52]. Probably, the mechanism for such association is due to milk-forming proteins (lactalbumin and casein), which have a uricosuric effect [53].

There is an inverse relation between coffee intake and UA concentrations [48], but such association seems to be due to other substances, as it does not seem to result from caffeine. Decreased UA concentrations would result from the improved insulin resistance observed with increased coffee intake, which occurs mainly due to chlorogenic acid, an antioxidant found in that drink [54,55].

Tsunoda *et al*. (2002) [56] observed the effects of hypocaloric diets on uric acid in overweight individuals. This type of diet indirectly decreased UA serum concentrations because it improved insulin sensitivity in these individuals, and this occurred regardless of changes in body weight or blood pressure.

## Uricemia as a risk factor

### Gouty arthritis

Gout is caused by articulation deposition of monosodium urate crystals after chronic hyperuricemia [2]. It affects 1-2% of adults in developed countries [2] and is considered an independent risk factor for cardiovascular disease [3]. Uric acid values higher than 7.0 mg/dl is a risk factor for the development of gout [3].

### Renal calculi

Renal calculi are formed by calcium salts deposited on an organic matrix of oxalate or urate. Their formation occurs in the glomerular filtrate in acid (oxalate or urate) or alkaline (carbonates and phosphates) pH in the presence of high calciuria (hyperparathyroidism or bone demineralization). They are usually formed and then dissolved, but under abnormal pH variation or filtrate concentration conditions, they may remain until excretion [30].

The contribution of UA to renal stone formation has an important role in progressive renal failure [57].

### Metabolic syndrome and body composition

Meta-analysis has recently shown that UA elevation is related to an increase in (coronary artery disease) infarction and mortality events [1], and epidemiological studies showed that UA is an independent risk factor for cardiovascular diseases [5,10], particularly in hypertensive and diabetic individuals [21]. It is also speculated that UA is one of the determinants of the metabolic syndrome [22]. Individuals with high UA levels have an odds ratio of 1.6-fold higher for developing MS [58].

UA is associated with Metabolic Syndrome [59] and its components [60], obesity, dyslipidemia, hypertension [18], insulin resistance (metabolic syndrome), increased C-reactive protein (PCR) concentration [23] and endothelial dysfunction [20], or even, to risk factors for cardiovascular diseases [7,17,19].

UA increase is observed in individuals with insulin resistance, probably because hyperisulinemia would cause lower renal UA excretion [61]. Additionally, insulin could indirectly act on UA, since there is an association between hyperinsulinemia and hypertriglyceridemia.

Some studies show that high concentrations of plasma triglycerides are related to hyperuricemia [58,62-64]. There are some explanations for such relation, and one of them is that during triglycerides (TG) synthesis there would be a greater need for NADPH [62]. The synthesis of fatty acids in the liver is associated with the *de novo *synthesis of purine, thus accelerating UA production [65].

Chen *et al*. (2007) [58] observed negative correlation between HDL-c and UA. The likely mechanism is the relationship between decreased HDL-C and insulin resistance [66]. Confirming this hypothesis, our research group showed that when adjusted for the other MS components, the relation between UA and HDL-c is lost [67].

In the adipose tissue, there is adipokine production, including that of leptin. One possible explanation for the association between higher waist circumference and hyperuricemia were suggested by Bedir et al. (2003) [68] and Fruehwald-Schultes et al. (1999) [69] which studies found that UA serum concentrations are independently related to leptin concentration, thus suggesting that would be a pathogenic factor responsible for UA increase in obese patients [58].

Some authors report UA to be responsible for blood pressure increase, and others show arterial hypertension (AH) to be a risk factor for hiperuricemia. UA inhibits the bioavailability of nitric oxide, which is a vasodilator [70], and, on the other hand, AH would lead to vascular disease and increase renal vascular resistance. Both of these processes reduce renal flow, thus increasing urate resorption [71].

A recent study showed an inverse relation between muscle mass (MM) and UA in healthy individuals older than 40 years [72]. Chronic elevation of UA concentrations would be a causal factor for sarcopenia, especially through increased inflammation and oxidative stress [72,73]. The activation of the xanthine oxidase metabolic pathway, which increases UA production and the superoxide radical [74] could elevate the reactive oxygen species (ROS) and it could be the main mechanism for the reduction of MM. Furthermore, UA exerts a pro-inflammatory effect, thus stimulating the production of interleukin-1, interleukin-6 the tumor necrosis factor which also can influence the muscle mass [75].

## Uricemia as a protective factor

It is not known whether UA would be a causal factor or an antioxidant protective response against oxidative stress [76]. While chronic high UA concentrations are associated to increased risk for CAD, acute elevations seem to provide antioxidant protection [77]. UA contributes to > 50% of the antioxidant capacity of the blood [78]. Furthermore, the UA has a protective action in vitamins C and E [79] with the stabilizing activities in these vitamins [79,80] and the presence of ascorbic acid in plasma is required for the antioxidant effect of UA [80]. Also, it has a direct effect on the inhibition of free radicals such as peroxyl radical and peroxynitrite, protecting the cell membrane and DNA [81,82].

Most authors do not consider UA as a detrimental factor to the body health, because of its antioxidant function [8,83]. The antioxidant activity of UA also occurs in the brain [84], being a protector for several disease such as multiple sclerosis and neurodegenerative disease. Higher concentration of UA is associated with lower risk of development of Parksons disease and a favorable effect at the disease progression mainly [85].

## Conclusions

**Figure 1:**
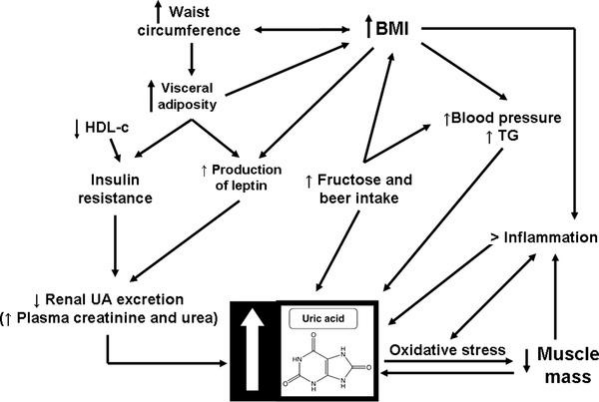
**Determinants of hyperuricemia**. UA = Uric Acid, BMI = Body Mass Index, TG = triglycerides.

Several factors are associated as cause and consequences of high UA concentration. Higher waist circumference and BMI are associated with higher insulin resistance and leptin production, and both reduce renal uric acid excretion, thus increasing its concentration. HDL-c concentration is negatively associated to insulin resistance, what can influence its negative correlation to uric acid. Obese individuals usually have Metabolic Syndrome diagnostic, which can also increase uric acid serum concentrations due to synthesis increase (triglycerides - TG concentration) and lower excretion (arterial hypertension - AH) (Figure 1).

Additionally, obesity and muscle mass (MM) reduction are associated with low-intensity chronic inflammation, and uric acid levels can increase in order to protect the organism against the moderate oxidative stress resulting from this situation. Low muscle mass (sarcopenia) is negatively associated with uric acid, However, it has not yet been clarified what the cause or effect is. Probably, oxidative stress produced by excessive uric acid can influence muscle mass reduction. The role played by diet on hyperuricemia has not yet been fully clarified, but high intake of fructose-rich industrialized food and high alcohol intake (particularly beer) seem to influence uricemia. The main mechanism of excretion of urate concentrations occurs by means of renal excretion; hence, glomerular function markers (urea and creatinine) are positively associated with uric acid (Figure 1).

Furthermore, there is still no consensus if UA is a protective or a risk factor, however, it seems that the quantity and the duration of the concentration of the uric acid in the blood is essential for this answer. Acute elevation seems to be a protective factor, whereas chronic elevation a risk factor.

## Competing interests

The authors declare that they have no competing interests.

## Authors' contributions

EPO wrote the manuscript, RCB read and corrected the final version of the manuscript. All authors read and approved.

## References

[B1] Kim SY Guevara JP Kim KM Choi HK Heitjan DF Albert DA Hyperuricemia and risk of stroke: a systematic review and meta-analysis Arthritis Rheum 2009 61 885 892 10.1002/art.24612 19565556 PMC2714267

[B2] Richette P Bardin T Gout Lancet 2010 375 318 328 10.1016/S0140-6736(09)60883-7 19692116

[B3] Roddy E Doherty M Epidemiology of gout Arthritis Res Ther 2010 12 223 10.1186/ar3199 21205285 PMC3046529

[B4] Ruggiero C Cherubini A Ble A Bos AJ Maggio M Dixit VD Lauretani F Bandinelli S Senin U Ferrucci L Uric acid and inflammatory markers Eur Heart J 2006 27 1174 1181 16611671 10.1093/eurheartj/ehi879 PMC2668163

[B5] Chen JH Chuang SY Chen HJ Yeh WT Pan WH Serum uric acid level as an independent risk factor for all-cause, cardiovascular, and ischemic stroke mortality: a Chinese cohort study Arthritis Rheum 2009 61 225 232 10.1002/art.24164 19177541

[B6] Hochberg MC Smolen JS Weinblatt ME Rheumatology 2003 3 New York: Mosby

[B7] Gagliardi AC Miname MH Santos RD Uric acid: A marker of increased cardiovascular risk Atherosclerosis 2009 202 11 17 10.1016/j.atherosclerosis.2008.05.022 18585721

[B8] Johnson RJ Kang DH Feig D Kivlighn S Kanellis J Watanabe S Tuttle KR Rodriguez-Iturbe B Herrera-Acosta J Mazzali M Is there a pathogenetic role for uric acid in hypertension and cardiovascular and renal disease? Hypertension 2003 41 1183 1190 10.1161/01.HYP.0000069700.62727.C5 12707287

[B9] Nakagawa T Tuttle KR Short RA Johnson RJ Hypothesis: fructose-induced hyperuricemia as a causal mechanism for the epidemic of the metabolic syndrome Nat Clin Pract Nephrol 2005 1 80 86 16932373 10.1038/ncpneph0019

[B10] Takahashi MM de Oliveira EP de Carvalho AL Dantas LA Burini FH Portero-McLellan KC Burini RC Metabolic Syndrome and dietary components are associated with coronary artery disease risk score in free-living adults: a cross-sectional study Diabetol Metab Syndr 2011 3 7 10.1186/1758-5996-3-7 21554698 PMC3103417

[B11] Roch-Ramel F Guisan B Renal Transport of Urate in Humans News Physiol Sci 1999 14 80 84 11390825 10.1152/physiologyonline.1999.14.2.80

[B12] Alvarez-Lario B Macarron-Vicente J Uric acid and evolution Rheumatology (Oxford) 2010 49 2010 2015 10.1093/rheumatology/keq204 20627967

[B13] Maxwell SR Thomason H Sandler D Leguen C Baxter MA Thorpe GH Jones AF Barnett AH Antioxidant status in patients with uncomplicated insulin-dependent and non-insulin-dependent diabetes mellitus Eur J Clin Invest 1997 27 484 490 10.1046/j.1365-2362.1997.1390687.x 9229228

[B14] Sautin YY Johnson RJ Uric acid: the oxidant-antioxidant paradox Nucleosides Nucleotides Nucleic Acids 2008 27 608 619 10.1080/15257770802138558 18600514 PMC2895915

[B15] Liebman SE Taylor JG Bushinsky DA Uric acid nephrolithiasis Curr Rheumatol Rep 2007 9 251 257 10.1007/s11926-007-0040-z 17531180

[B16] Terkeltaub R Bushinsky DA Becker MA Recent developments in our understanding of the renal basis of hyperuricemia and the development of novel antihyperuricemic therapeutics Arthritis Res Ther 2006 8 Suppl 1 S4 10.1186/ar1909 16820043 PMC3226109

[B17] Burack RC Keller JB Higgins MW Cardiovascular risk factors and obesity: are baseline levels of blood pressure, glucose, cholesterol and uric acid elevated prior to weight gain? J Chronic Dis 1985 38 865 872 10.1016/0021-9681(85)90111-0 4044772

[B18] Doehner W Schoene N Rauchhaus M Leyva-Leon F Pavitt DV Reaveley DA Schuler G Coats AJ Anker SD Hambrecht R Effects of xanthine oxidase inhibition with allopurinol on endothelial function and peripheral blood flow in hyperuricemic patients with chronic heart failure: results from 2 placebo-controlled studies Circulation 2002 105 2619 2624 10.1161/01.CIR.0000017502.58595.ED 12045167

[B19] Fang J Alderman MH: Serum uric acid and cardiovascular mortality the NHANES I epidemiologic follow-up study, 1971-1992. National Health and Nutrition Examination Survey Jama 2000 283 2404 2410 10.1001/jama.283.18.2404 10815083

[B20] Farquharson CA Butler R Hill A Belch JJ Struthers AD Allopurinol improves endothelial dysfunction in chronic heart failure Circulation 2002 106 221 226 10.1161/01.CIR.0000022140.61460.1D 12105162

[B21] Feig DI Johnson RJ Hyperuricemia in childhood primary hypertension Hypertension 2003 42 247 252 10.1161/01.HYP.0000085858.66548.59 12900431 PMC1800942

[B22] Onat A Uyarel H Hergenc G Karabulut A Albayrak S Sari I Yazici M Keles I Serum uric acid is a determinant of metabolic syndrome in a population-based study Am J Hypertens 2006 19 1055 1062 10.1016/j.amjhyper.2006.02.014 17027827

[B23] Saito M Ishimitsu T Minami J Ono H Ohrui M Matsuoka H Relations of plasma high-sensitivity C-reactive protein to traditional cardiovascular risk factors Atherosclerosis 2003 167 73 79 10.1016/S0021-9150(02)00380-5 12618270

[B24] Mount DB Kwon CY Zandi-Nejad K Renal urate transport Rheum Dis Clin North Am 2006 32 313 331 vi 10.1016/j.rdc.2006.02.006 16716882

[B25] Shima Y Teruya K Ohta H Association between intronic SNP in urate-anion exchanger gene, SLC22A12, and serum uric acid levels in Japanese Life Sci 2006 79 2234 2237 10.1016/j.lfs.2006.07.030 16920156

[B26] Ejaz AA Mu W Kang DH Roncal C Sautin YY Henderson G Tabah-Fisch I Keller B Beaver TM Nakagawa T Johnson RJ Could uric acid have a role in acute renal failure? Clin J Am Soc Nephrol 2007 2 16 21 17699382 10.2215/CJN.00350106

[B27] Becker BF Towards the physiological function of uric acid Free Radic Biol Med 1993 14 615 631 10.1016/0891-5849(93)90143-I 8325534

[B28] Manfredi JP Holmes EW Purine salvage pathways in myocardium Annu Rev Physiol 1985 47 691 705 10.1146/annurev.ph.47.030185.003355 2986542

[B29] Watts RW Uric acid production with particular reference to the role of xanthine oxidase and its inhibition Proc R Soc Med 1966 59 287 292 5937675 10.1177/003591576605900401 PMC1900614

[B30] Hediger MA Johnson RJ Miyazaki H Endou H Molecular physiology of urate transport Physiology (Bethesda) 2005 20 125 133 10.1152/physiol.00039.2004 15772301

[B31] Wu XW Muzny DM Lee CC Caskey CT Two independent mutational events in the loss of urate oxidase during hominoid evolution J Mol Evol 1992 34 78 84 10.1007/BF00163854 1556746

[B32] Yu KH See LC Huang YC Yang CH Sun JH Dietary factors associated with hyperuricemia in adults Semin Arthritis Rheum 2008 37 243 250 10.1016/j.semarthrit.2007.04.007 17570471

[B33] Emmerson BT The management of gout N Engl J Med 1996 334 445 451 10.1056/NEJM199602153340707 8552148

[B34] Yu T Yu TF Milestones in the treatment of gout Am J Med 1974 56 676 685 10.1016/0002-9343(74)90634-2 4604776

[B35] Cuppari L Guia de nutrição: nutrição clínica no adulto 2005 2 Barueri: Manole 474

[B36] Choi HK Liu S Curhan G Intake of purine-rich foods, protein, and dairy products and relationship to serum levels of uric acid: the Third National Health and Nutrition Examination Survey Arthritis Rheum 2005 52 283 289 10.1002/art.20761 15641075

[B37] Champe PC Harvey RA Biochemistry 1994 2 Philadelphia: J.B. Lippincott: Lippincotts' illustrated reviews 253 255

[B38] Wrolstad RE Shallenberger RS Free sugars and sorbitol in fruits-a complication from the literature J Assoc Off Anal Chem 1981 64 91 103 7204315

[B39] Bode JC Zelder O Rumpelt HJ Wittkamp U Depletion of liver adenosine phosphates and metabolic effects of intravenous infusion of fructose or sorbitol in man and in the rat Eur J Clin Invest 1973 3 436 441 10.1111/j.1365-2362.1973.tb02211.x 4772339

[B40] Lotito SB Frei B Consumption of flavonoid-rich foods and increased plasma antioxidant capacity in humans: cause, consequence, or epiphenomenon? Free Radic Biol Med 2006 41 1727 1746 10.1016/j.freeradbiomed.2006.04.033 17157175

[B41] Hallfrisch J Metabolic effects of dietary fructose Faseb J 1990 4 2652 2660 2189777 10.1096/fasebj.4.9.2189777

[B42] Mayes PA Intermediary metabolism of fructose Am J Clin Nutr 1993 58 754S 765S 8213607 10.1093/ajcn/58.5.754S

[B43] Nakagawa T Hu H Zharikov S Tuttle KR Short RA Glushakova O Ouyang X Feig DI Block ER Herrera-Acosta J A causal role for uric acid in fructose-induced metabolic syndrome Am J Physiol Renal Physiol 2006 290 F625 F631 16234313 10.1152/ajprenal.00140.2005

[B44] Choi HK Gao X Curhan G Vitamin C intake and the risk of gout in men: a prospective study Arch Intern Med 2009 169 502 507 10.1001/archinternmed.2008.606 19273781 PMC2767211

[B45] Berger L Gerson CD Yu TF The effect of ascorbic acid on uric acid excretion with a commentary on the renal handling of ascorbic acid Am J Med 1977 62 71 76 10.1016/0002-9343(77)90351-5 835593

[B46] Stein HB Hasan A Fox IH Ascorbic acid-induced uricosuria. A consequency of megavitamin therapy Ann Intern Med 1976 84 385 388 1259282 10.7326/0003-4819-84-4-385

[B47] Gibson T Rodgers AV Simmonds HA Toseland P Beer drinking and its effect on uric acid Br J Rheumatol 1984 23 203 209 10.1093/rheumatology/23.3.203 6743968

[B48] Choi HK Curhan G Coffee, tea, and caffeine consumption and serum uric acid level: the third national health and nutrition examination survey Arthritis Rheum 2007 57 816 821 10.1002/art.22762 17530681

[B49] Drum DE Goldman PA Jankowski CB Elevation of serum uric acid as a clue to alcohol abuse Arch Intern Med 1981 141 477 479 10.1001/archinte.1981.00340040073020 7212890

[B50] Eastmond CJ Garton M Robins S Riddoch S The effects of alcoholic beverages on urate metabolism in gout sufferers Br J Rheumatol 1995 34 756 759 10.1093/rheumatology/34.8.756 7551661

[B51] Faller J Fox IH Ethanol-induced hyperuricemia: evidence for increased urate production by activation of adenine nucleotide turnover N Engl J Med 1982 307 1598 1602 10.1056/NEJM198212233072602 7144847

[B52] Choi HK Atkinson K Karlson EW Willett W Curhan G Purine-rich foods, dairy and protein intake, and the risk of gout in men N Engl J Med 2004 350 1093 1103 10.1056/NEJMoa035700 15014182

[B53] Ghadirian P Shatenstein B Verdy M Hamet P The influence of dairy products on plasma uric acid in women Eur J Epidemiol 1995 11 275 281 10.1007/BF01719431 7493659

[B54] Arion WJ Canfield WK Ramos FC Schindler PW Burger HJ Hemmerle H Schubert G Below P Herling AW Chlorogenic acid and hydroxynitrobenzaldehyde: new inhibitors of hepatic glucose 6-phosphatase Arch Biochem Biophys 1997 339 315 322 10.1006/abbi.1996.9874 9056264

[B55] Wu T Giovannucci E Pischon T Hankinson SE Ma J Rifai N Rimm EB Fructose, glycemic load, and quantity and quality of carbohydrate in relation to plasma C-peptide concentrations in US women Am J Clin Nutr 2004 80 1043 1049 15447918 10.1093/ajcn/80.4.1043

[B56] Tsunoda S Kamide K Minami J Kawano Y Decreases in serum uric acid by amelioration of insulin resistance in overweight hypertensive patients: effect of a low-energy diet and an insulin-sensitizing agent Am J Hypertens 2002 15 697 701 10.1016/S0895-7061(02)02953-9 12160192

[B57] Capasso G Jaeger P Robertson WG Unwin RJ Uric acid and the kidney: urate transport, stone disease and progressive renal failure Curr Pharm Des 2005 11 4153 4159 10.2174/138161205774913219 16375737

[B58] Chen LY Zhu WH Chen ZW Dai HL Ren JJ Chen JH Chen LQ Fang LZ Relationship between hyperuricemia and metabolic syndrome J Zhejiang Univ Sci B 2007 8 593 598 17657863 10.1631/jzus.2007.B0593 PMC1934956

[B59] Sui X Church TS Meriwether RA Lobelo F Blair SN Uric acid and the development of metabolic syndrome in women and men Metabolism 2008 57 845 852 10.1016/j.metabol.2008.01.030 18502269 PMC2486830

[B60] Abdullah AR Hasan HA Raigangar VL Analysis of the Relationship of Leptin, High-Sensitivity C-Reactive Protein, Adiponectin, Insulin, and Uric Acid to Metabolic Syndrome in Lean, Overweight, and Obese Young Females Metab Syndr Relat Disord 2009 7 17 22 10.1089/met.2008.0045 19025443

[B61] Facchini F Chen YD Hollenbeck CB Reaven GM Relationship between resistance to insulin-mediated glucose uptake, urinary uric acid clearance, and plasma uric acid concentration Jama 1991 266 3008 3011 10.1001/jama.1991.03470210076036 1820474

[B62] Clausen JO Borch-Johnsen K Ibsen H Pedersen O Analysis of the relationship between fasting serum uric acid and the insulin sensitivity index in a population-based sample of 380 young healthy Caucasians Eur J Endocrinol 1998 138 63 69 10.1530/eje.0.1380063 9461318

[B63] Conen D Wietlisbach V Bovet P Shamlaye C Riesen W Paccaud F Burnier M Prevalence of hyperuricemia and relation of serum uric acid with cardiovascular risk factors in a developing country BMC Public Health 2004 4 9 10.1186/1471-2458-4-9 15043756 PMC406506

[B64] Schachter M Uric acid and hypertension Curr Pharm Des 2005 11 4139 4143 10.2174/138161205774913246 16375735

[B65] Matsuura F Yamashita S Nakamura T Nishida M Nozaki S Funahashi T Matsuzawa Y Effect of visceral fat accumulation on uric acid metabolism in male obese subjects: visceral fat obesity is linked more closely to overproduction of uric acid than subcutaneous fat obesity Metabolism 1998 47 929 933 10.1016/S0026-0495(98)90346-8 9711987

[B66] Schmidt MI Watson RL Duncan BB Metcalf P Brancati FL Sharrett AR Davis CE Heiss G Clustering of dyslipidemia, hyperuricemia, diabetes, and hypertension and its association with fasting insulin and central and overall obesity in a general population. Atherosclerosis Risk in Communities Study Investigators Metabolism 1996 45 699 706 10.1016/S0026-0495(96)90134-1 8637443

[B67] de Oliveira EP Manda RM Torezan GA Corrente JE Burini RC Dietary, anthropometric, and biochemical determinants of plasma high-density lipoprotein-cholesterol in free-living adults Cholesterol 2011 2011 851750 21490775 10.1155/2011/851750 PMC3065868

[B68] Bedir A Topbas M Tanyeri F Alvur M Arik N Leptin might be a regulator of serum uric acid concentrations in humans Jpn Heart J 2003 44 527 536 10.1536/jhj.44.527 12906034

[B69] Fruehwald-Schultes B Peters A Kern W Beyer J Pfutzner A Serum leptin is associated with serum uric acid concentrations in humans Metabolism 1999 48 677 680 10.1016/S0026-0495(99)90163-4 10381138

[B70] Baldus S Koster R Chumley P Heitzer T Rudolph V Ostad MA Warnholtz A Staude HJ Thuneke F Koss K Oxypurinol improves coronary and peripheral endothelial function in patients with coronary artery disease Free Radic Biol Med 2005 39 1184 1190 10.1016/j.freeradbiomed.2005.06.004 16214034 PMC2170895

[B71] Messerli FH Frohlich ED Dreslinski GR Suarez DH Aristimuno GG Serum uric acid in essential hypertension: an indicator of renal vascular involvement Ann Intern Med 1980 93 817 821 7447188 10.7326/0003-4819-93-6-817

[B72] Beavers KM Beavers DP Serra MC Bowden RG Wilson RL Low relative skeletal muscle mass indicative of sarcopenia is associated with elevations in serum uric acid levels: Findings from NHANES III Journal of Nutrition, Health and Aging 2009 13 177 182 10.1007/s12603-009-0054-5 PMC12876367 19262948

[B73] Pierine DT Nicola M de Oliveira EP Sarcopenia: alterações metabólicas e consequências no envelhecimento R bras Ci e Mov 2009 17 96 103

[B74] Powers SK Kavazis AN DeRuisseau KC Mechanisms of disuse muscle atrophy: role of oxidative stress Am J Physiol Regul Integr Comp Physiol 2005 288 R337 R344 15637170 10.1152/ajpregu.00469.2004

[B75] Kanellis J Kang DH Uric acid as a mediator of endothelial dysfunction, inflammation, and vascular disease Semin Nephrol 2005 25 39 42 10.1016/j.semnephrol.2004.09.007 15660333

[B76] Baillie JK Bates MG Thompson AA Waring WS Partridge RW Schnopp MF Simpson A Gulliver-Sloan F Maxwell SR Webb DJ Endogenous urate production augments plasma antioxidant capacity in healthy lowland subjects exposed to high altitude Chest 2007 131 1473 1478 10.1378/chest.06-2235 17494796

[B77] Yu ZF Bruce-Keller AJ Goodman Y Mattson MP Uric acid protects neurons against excitotoxic and metabolic insults in cell culture, and against focal ischemic brain injury in vivo J Neurosci Res 1998 53 613 625 10.1002/(SICI)1097-4547(19980901)53:5<613::AID-JNR11>3.0.CO;2-1 9726432

[B78] Glantzounis GK Tsimoyiannis EC Kappas AM Galaris DA Uric acid and oxidative stress Curr Pharm Des 2005 11 4145 4151 10.2174/138161205774913255 16375736

[B79] Ma YS Stone WL LeClair IO The effects of vitamin C and urate on the oxidation kinetics of human low-density lipoprotein Proc Soc Exp Biol Med 1994 206 53 59 8183962 10.3181/00379727-206-43722

[B80] Frei B Stocker R Ames BN Antioxidant defenses and lipid peroxidation in human blood plasma Proc Natl Acad Sci USA 1988 85 9748 9752 10.1073/pnas.85.24.9748 3200852 PMC282858

[B81] Hooper DC Spitsin S Kean RB Champion JM Dickson GM Chaudhry I Koprowski H Uric acid, a natural scavenger of peroxynitrite, in experimental allergic encephalomyelitis and multiple sclerosis Proc Natl Acad Sci USA 1998 95 675 680 10.1073/pnas.95.2.675 9435251 PMC18479

[B82] Wayner DD Burton GW Ingold KU Barclay LR Locke SJ The relative contributions of vitamin E, urate, ascorbate and proteins to the total peroxyl radical-trapping antioxidant activity of human blood plasma Biochim Biophys Acta 1987 924 408 419 10.1016/0304-4165(87)90155-3 3593759

[B83] Kutzing MK Firestein BL Altered uric acid levels and disease states J Pharmacol Exp Ther 2008 324 1 7 17890445 10.1124/jpet.107.129031

[B84] Scott GS Hooper DC The role of uric acid in protection against peroxynitrite-mediated pathology Med Hypotheses 2001 56 95 100 10.1054/mehy.2000.1118 11133262

[B85] Cipriani S Chen X Schwarzschild MA Urate: a novel biomarker of Parkinson's disease risk, diagnosis and prognosis Biomark Med 2010 4 701 712 10.2217/bmm.10.94 20945982 PMC3049925

